# Do We Need to Monitor B‐Vitamins and Homocysteine During Initiation of Foslevodopa/Foscarbidopa Therapy?

**DOI:** 10.1002/mds.70388

**Published:** 2026-06-01

**Authors:** Aida Shaghaghi‐Zadeh, Matthias Höllerhage, Florian Wegner, Lan Ye, Ishana Schneidereit, Tobias Hegelmaier, Rebecca Jacob, Christoph Schrader, Martin Klietz

**Affiliations:** ^1^ Department of Neurology Hannover Medical School Hannover Germany; ^2^ Department of Psychiatry, Social Psychiatry and Psychotherapy Hannover Medical School Hannover Germany

**Keywords:** folate, foslevodopa infusion therapy, homocysteine, Parkinson's disease, vitamin B12, vitamin B6

Foslevodopa/foscarbidopa is a recently introduced subcutaneous infusion therapy for patients with advanced Parkinson's disease (PD) insufficiently controlled by conventional therapies.^1^, ^2^ As foslevodopa is a levodopa (l‐dopa) prodrug, its administration is directly linked to l‐dopa metabolism.^3^ In patients receiving l‐dopa therapy, including oral l‐dopa/carbidopa and l‐dopa/carbidopa intestinal gel, alterations in B‐vitamin metabolism and homocysteine levels have been reported.^4^ However, data on short‐term changes in B‐vitamin and homocysteine levels during initiation of foslevodopa/foscarbidopa infusion therapy remain limited.

We retrospectively included 18 patients with PD initiating continuous subcutaneous foslevodopa/foscarbidopa infusion therapy during inpatient treatment.

Plasma levels of vitamin B6, folate (vitamin B9), vitamin B12, and homocysteine were measured before therapy initiation and again before discharge after dose titration (mean interval: 8.1 ± 0.8 days, mean ± standard error of the mean). Patients starting vitamin supplementation between measurements were excluded from the respective paired analyses. Available paired laboratory data included vitamin B6 (n = 12), folate (n = 12), vitamin B12 (n = 15), and homocysteine (n = 15). Paired *t* tests were used for statistical analyses; *P* < 0.05 was considered significant. Demographic and clinical characteristics are summarized in Table S1.

Vitamin B6 levels decreased significantly from 21.02 ± 2.34 μg/L at baseline to 14.78 ± 1.38 μg/L at follow‐up (*P* = 0.012) (Fig. 1A). In contrast, folate levels did not change significantly (baseline 15.18 ± 3.91 μg/L vs. follow‐up 14.26 ± 3.77 μg/L; *P* = 0.212) (Fig. 1B). No significant difference was observed for vitamin B12 levels (baseline 497.00 ± 40.23 ng/L vs. follow‐up 484.20 ± 30.19 ng/L; *P* = 0.474) (Fig. 1C). Homocysteine levels increased significantly from 16.48 ± 2.06 μmol/L at baseline to 18.71 ± 2.16 μmol/L at follow‐up (*P* = 0.048) (Fig. 1D).

**FIG. 1:**
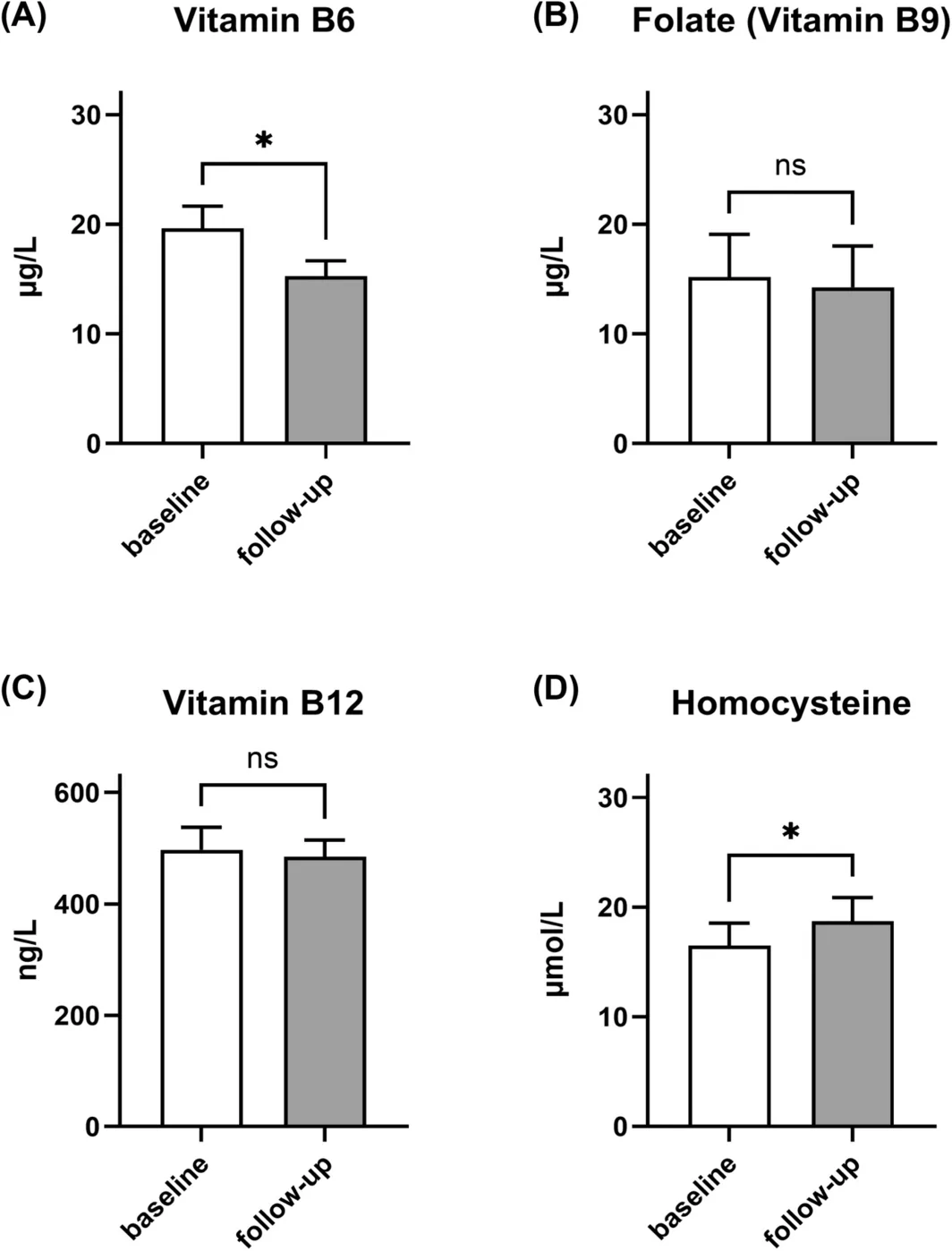
Plasma levels of vitamin B6, folate (B9) B12, and homocysteine at baseline and after initiation of foslevodopa/foscarbidopa infusion therapy. **(A)** Vitamin B6 data are presented as mean ± SEM (standard error of the mean, n = 12). Baseline mean was 21.02 ± 2.34 μg/L, and after 8 days follow‐up mean was 14.78 ± 1.38 μg/L. A paired *t* test showed a significant decrease (*P = 0.012). **(B)** Folate (vitamin B9) data are presented as mean ± SEM (n = 12). Baseline mean was 15.18 ± 3.91 μg/L, and follow‐up mean was 14.26 ± 3.77 μg/L. No significant change was observed (*P* = 0.212). **(C)** Vitamin B12 data are presented as mean ± SEM (n = 15). Baseline mean was 497.00 ± 40.23 pg/mL, and follow‐up mean was 484.20 ± 30.19 pg/mL. No significant change was observed (*P* = 0.474). **(D)** Homocysteine data are presented as mean ± SEM (n = 15). Baseline mean was 16.48 ± 2.06 μmol/L, and after 8 days follow‐up mean was 18.71 ± 2.16 μmol/L. A paired *t* test showed a significant increase (*P = 0.048). Baseline values are shown in blue and follow‐up values after 8 days in orange. *P < 0.05 was considered statistically significant.

The changes in vitamin B6 and homocysteine are likely explained by the high and continuous l‐dopa exposure delivered by the foslevodopa/foscarbidopa infusion therapy.^5^ l‐Dopa metabolism involves B‐vitamins that act as cofactors in homocysteine metabolism. In this context, COMT‐mediated l‐dopa metabolism increases homocysteine, whereas its clearance depends on pyridoxal‐5′‐phosphate–dependent transsulfuration, explaining the concomitant homocysteine increase and vitamin B6 reduction.^6^ These findings are consistent with a recent case series linking foslevodopa/foscarbidopa therapy to axonal polyneuropathy, with vitamin deficiency and hyperhomocysteinemia as contributing factors.^7^

This study is the first to systematically demonstrate that initiation of foslevodopa/foscarbidopa infusion therapy in advanced PD is associated with a rapid and significant reduction in vitamin B6 levels and an increase in homocysteine levels, whereas folate and vitamin B12 levels remain stable. Due to the mean follow‐up interval of ~8 days, no early decline in vitamin B12 levels would necessarily be expected, and the absence of a significant change should therefore be interpreted cautiously. These findings highlight the need to monitor B6 and homocysteine during foslevodopa/foscarbidopa infusion therapy, particularly in patients with preexisting low vitamin B6 levels, for whom early supplementation may be clinically relevant. The main limitation of this study is the limited number of patients. Larger studies are warranted.

## Author Roles

(1) Research project: A. Conception, B. Organization, C. Execution; (2) Statistical analysis: A. Design, B. Execution, C. Review and critique; (3) Manuscript preparation: A. Writing of the first draft, B. Review and critique.

A.S.‐Z.: 1B, 1C, 2A, 2B, 3A

M.H.: 1A, 1B, 2A, 3B

F.W.: 1C, 2C, 3B

L.Y.: 1C, 2C, 3B

I.S.: 1C, 2C, 3B

T.H.: 1C, 2C, 3B

R.J.: 1C, 2C, 3B

C.S.: 1C, 2C, 3B

M.K.: 1A, 1B, 2A, 2C, 3A, 3B

## Financial Disclosures and Conflicts of Interest

The authors do not have any relevant disclosures concerning this publication.

## Supporting information


**Table S1.** Demographics and patient characteristics.

## Data Availability

The data that support the findings of this study are available on request from the corresponding author. The data are not publicly available due to privacy or ethical restrictions.
