# Supplementary material for: Do We Need to Monitor B‐Vitamins and Homocysteine During Initiation of Foslevodopa/Foscarbidopa Therapy?
Source: Mov Disord. 2026 Jun 1;41(9):2535–7. doi: 10.1002/mds.70388 (PMC13602287; doi:10.1002/mds.70388)
Supplement: Supplementary file 1 [file MDS-41-2535-s001.docx]

**Supplementary table 1**

**Demographics and patient characteristics**

Sex, male/female: 9/6 (60%/40%)

|  | Mean | SD | Min | Max |
| --- | --- | --- | --- | --- |
| Age (years) | 67.0 | 8.5 | 52 | 80 |
| Disease duration (years) | 13.0 | 5.8 | 6 | 29 |
| Hoehn and Yahr stage | 2.9 | 0.8 | 2 | 4 |
| MDS-UPDRS III | 32.7 | 12.3 | 14 | 54 |
| MoCA | 24.1 | 3.8 | 19 | 29 |
| GDS-15 | 4.7 | 2.7 | 2 | 9 |
| LED at baseline (mg) | 1272.9 | 515.0 | 535 | 2400 |
| LED at discharge (mg) | 1515.3 | 398.5 | 856.8 | 2160.4 |

Abbreviations: MDS-UPDRS III Movement Disorders Society sponsored revision of the Unified Parkinson’s Disease Rating Scale part III; MoCA Montreal Cognitive Assessment; GDS-15 Geriatric Depression Scale-15; LED Levodopa Equivalent Dose.
